# Intracellular Targeting Specificity of Novel Phthalocyanines Assessed in a Host-Parasite Model for Developing Potential Photodynamic Medicine

**DOI:** 10.1371/journal.pone.0020786

**Published:** 2011-06-06

**Authors:** Sujoy Dutta, Benson G. Ongarora, Hairong Li, Maria da Graca H. Vicente, Bala K. Kolli, Kwang Poo Chang

**Affiliations:** 1 Department of Microbiology/Immunology, Chicago Medical School/Rosalind Franklin University of Medicine and Science, North Chicago, Illinois, United States of America; 2 Department of Chemistry, Louisiana State University, Baton Rouge, Louisiana, United States of America; University of Georgia, United States of America

## Abstract

Photodynamic therapy, unlikely to elicit drug-resistance, deserves attention as a strategy to counter this outstanding problem common to the chemotherapy of all diseases. Previously, we have broadened the applicability of this modality to photodynamic vaccination by exploiting the unusual properties of the trypanosomatid protozoa, *Leishmania*, i.e., their innate ability of homing to the phagolysosomes of the antigen-presenting cells and their selective photolysis therein, using transgenic mutants endogenously inducible for porphyrin accumulation. Here, we extended the utility of this host-parasite model for *in vitro* photodynamic therapy and vaccination by exploring exogenously supplied photosensitizers. Seventeen novel phthalocyanines (Pcs) were screened *in vitro* for their photolytic activity against cultured *Leishmania*. Pcs rendered cationic and soluble (csPcs) for cellular uptake were phototoxic to both parasite and host cells, i.e., macrophages and dendritic cells. The csPcs that targeted to mitochondria were more photolytic than those restricted to the endocytic compartments. Treatment of infected cells with endocytic csPcs resulted in their accumulation in *Leishmania*-containing phagolysosomes, indicative of reaching their target for photodynamic therapy, although their parasite versus host specificity is limited to a narrow range of csPc concentrations. In contrast, *Leishmania* pre-loaded with csPc were selectively photolyzed intracellularly, leaving host cells viable. Pre-illumination of such csPc-loaded *Leishmania* did not hinder their infectivity, but ensured their intracellular lysis. Ovalbumin (OVA) so delivered by photo-inactivated OVA transfectants to mouse macrophages and dendritic cells were co-presented with MHC Class I molecules by these antigen presenting cells to activate OVA epitope-specific CD8+T cells. The *in vitro* evidence presented here demonstrates for the first time not only the potential of endocytic csPcs for effective photodynamic therapy against *Leishmania* but also their utility in photo-inactivation of *Leishmania* to produce a safe carrier to express and deliver a defined antigen with enhanced cell-mediated immunity.

## Introduction

Photodynamic therapy (PT) eliminates diseased cells/pathogens by using photosensitizers (PS) that are excitable by light to produce cytotoxic reactive oxygen species (ROS) in the presence of oxygen [1]. Since the ROS simultaneously attack multiple molecules of very different properties, PT is considered to have the potential to circumvent the problem of drug-resistance common to both infectious [2] and non-infectious diseases [3], [4].

Recently, PT has been explored for treating clinical and experimental cutaneous leishmaniasis [5]–[12]. The causative agents of this vector-borne, zoonotic disease are trypanosomatid protozoa of *Leishmania spp.*, which is wide-spread, having an annual incidence of ∼2 million cases in ∼90 countries, putting a worldwide population of 350 million at risk [13]. Effective drugs have never been developed specifically for this and related diseases, i. e. the debilitating mucocutaneous leishmaniasis and the often fatal visceral leishmaniasis. As expected, resistance has developed from the continuous use of ineffective drugs, e. g. pentavalent antimony [14]. Consequently, clinical management of these diseases is difficult [15], [16], while vaccines are still under development [17], [18]. In natural infections, all pathogenic *Leishmania spp.* show the homing specificity to parasitize mononuclear phagocytes, e.g. macrophages (MC) and dendritic cells (DC) [19]–[21]. MCs are the exclusive host cells where *Leishmania* reside in their phagolysosomes [20]. How PS can be targeted to this site against *Leishmania* with specificity is a challenging issue.

While *Leishmania* is a potential target of therapeutic PT, it is also uniquely exploitable to facilitate PT against other diseases due to its unusual mechanism of parasitism in the MC phagolysosome. Attenuated *Leishmania* thus may be used as a carrier for delivery of drugs/vaccines to this site for activation or presentation to enhance their activities. Previously, we have obtained evidence for this by using uroporphyrin I (URO) as a potent leishmanolytic PS, which was induced endogenously for selective accumulation in transgenic mutants, but not in the host cells, for effective photodynamic vaccination [22], [23]. Photo-sensitization of *Leishmania* with exogenously supplied PS presents an alternative approach to achieve the same aim. Our previous studies along this line indicate that photolytic activity and specificity of PS is correlated with its cellular uptake and intracellular trafficking [7], [24]. Thus, while URO generated intracellularly is highly phototoxic, exogenously supplied URO is not taken up by cells and is thus photolytically inactive [24]. In contrast, exogenously supplied aluminum phthalocyanine chloride (AlPhCl) becomes associated with *Leishmania* rapidly and sensitized them for photolysis, but this is non-selective when sensitized parasites were used to infect the host cells, as the latter were also lysed [7]. These preliminary observations underscore the necessity of further investigation with additional PS to understand their structure-function relationships.

In the present study, 17 novel phthalocyanines (Pc) were examined for PT activities in our host-parasite *in vitro* model. Soluble cationic Pcs (csPcs), which were taken up by endocytosis or targeted to mitochondria, were found to mediate photolysis effectively. While both are photolytic to *Leishmania*, they differ in parasite versus host selectivity. From a series of *in vitro* studies, we obtained evidence, indicating that the endocytic csPcs are favorably disposed for PT against cutaneous leishmaniasis; and that csPc-loaded *Leishmania* photolytically delivers a model antigen to MCs and DCs for presentation to activate specific T cells, supportive of their carrier potential for immuno-prophylactic and –therapeutic PT.

## Results

### csPcs sensitize *Leishmania* and macrophages differentially for photolysis

**Figure 1:**
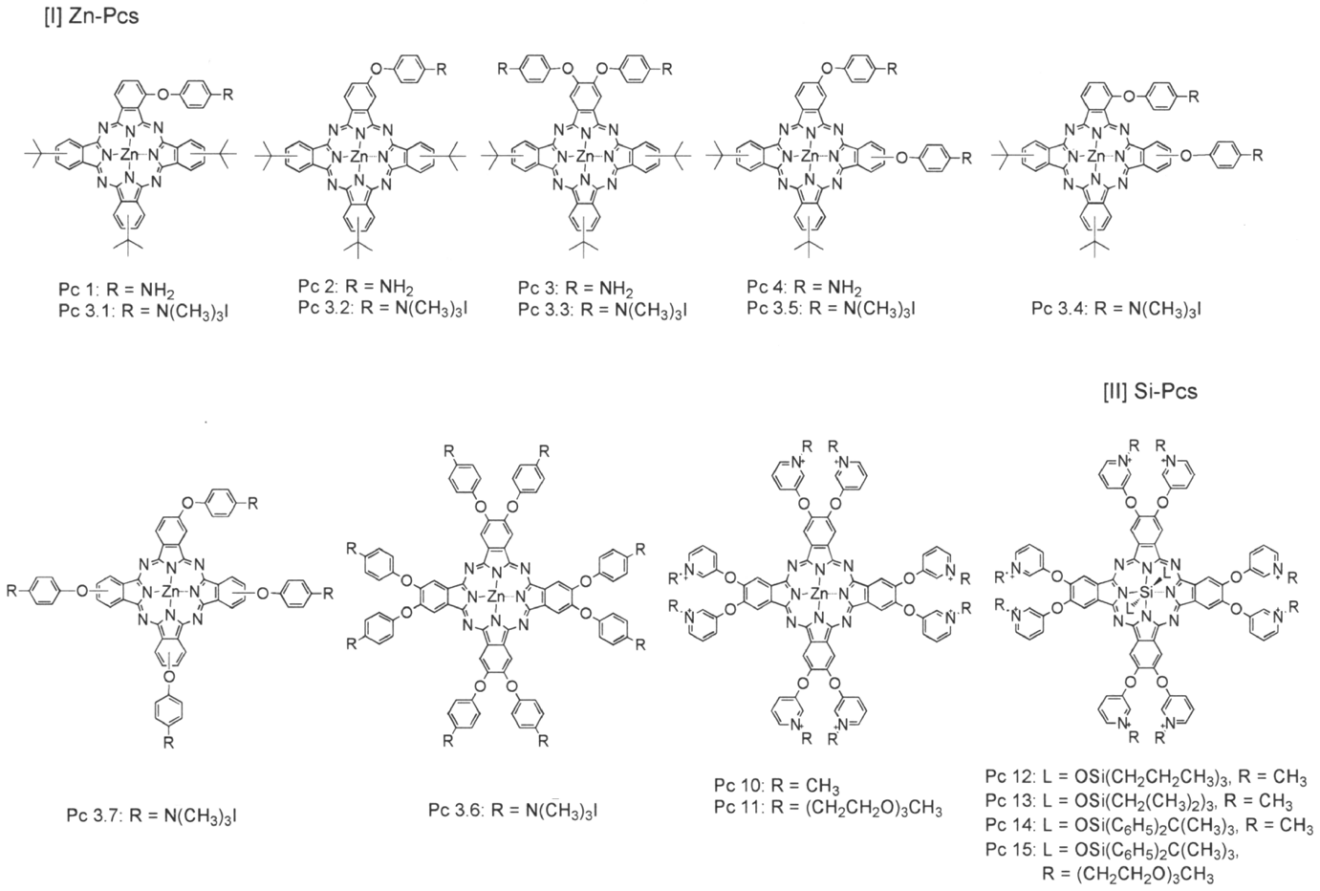
Structures of zinc-phthalocyanines (Zn-Pc) and silicon-phthalocyanines (Si-Pc) used in this study. **Pc 1–3.7**, anilinium phthalocyanines; **Pc 10–15**, pyridyloxy phthalocyanines.

**Figure 2:**
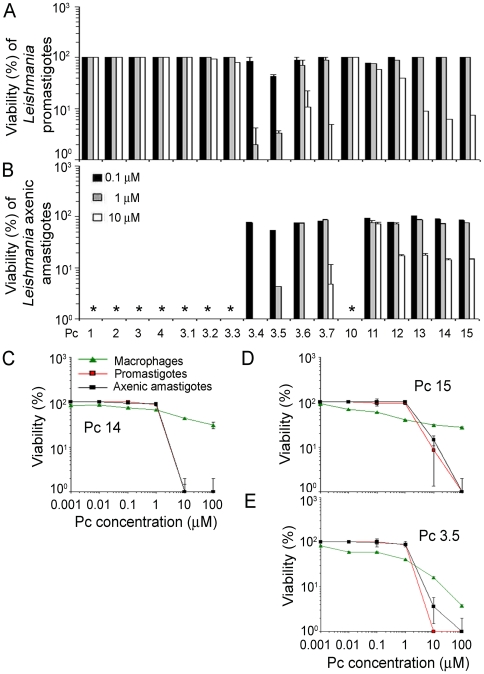
Photosensitivity of *L. amazonensis* and macrophages to different phthalocyanines. [**A**] Promastigotes and [**B**] axenic amastigotes were treated with and light-exposed in the presence of csPcs (0.1–10 µM) as indicated. **[C]Pc14, [D]Pc15, and [E]Pc3.5:** Promastigotes, axenic amastigotes and J774 macrophages were “pre-loaded” with the 3 csPcs as indicated and light-exposed as described. Cell viability was assessed 1-day later by MTT assays ([**A–E**]). * Not done.

All Pcs (**Figure 1**) were assessed initially at 3 different concentrations each (0.1–10 µM) against the promastigote stage (**Figure 2A**). Cell suspensions were first treated with Pcs in the dark overnight and then exposed to light at a fluence of 2.0 J/cm^2^ (referred to as light exposure hereafter). Of the 17 Pcs examined under these conditions, 9 produced phototoxic phenotypes, as seen microscopically as loss of cell motility and/or integrity. These phenotypes were not noticeable immediately but were observed after incubation for ∼16 hrs when cell viability was assessed by (3-(4,5-Dimethylthiazol-2-yl)-2,5-diphenyltetrazolium bromide (MTT) assays. Phototoxicity so determined was dose-dependent, but varied with the 9 effective Pcs. Pc 3.5 was most photolytic (**Figure 2A**), as indicated by the manifestation of cytolysis more rapidly than others after illumination (not shown). These 9 anti-promastigote Pcs were also found equally photolytic against the axenic amastigotes (**Figure 2B** **vs** **Figure 2A**). Notably, the effective Pcs are all cationic and soluble (csPc), i. e. 4 anilinium Pcs 3.4 to 3.7 and 5 pyridyloxy Pcs 11 to15 (see **Figure 1**).

**Figure 3:**
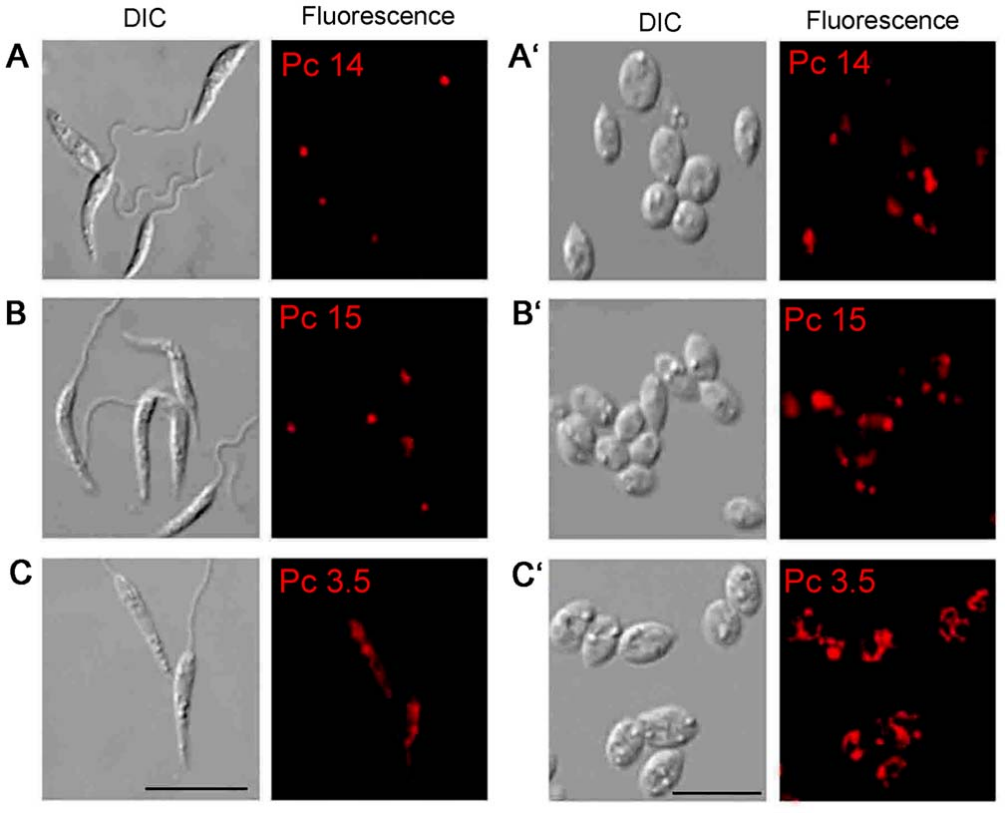
Uptake of csPcs by two different *Leishmania* stages. [**A–C**] Promastigotes and [**A′–C′**] axenic amastigotes were pre-loaded with 10 µM csPcs 14, 15 or 3.5, respectively. **DIC**, Differential interference. **Fluorescence**, Pc intracellular fluorescence. Scale bar = 10 µm.

Three most effective csPcs of the 2 chemical series, i. e. Pcs 3.5, and 14/15, were further studied. Both *Leishmania* stages and their MC host cells were exposed separately to these csPcs in serial dilutions (0.001 to 100 µM) and washed to remove non-cell associated Pcs (referred to as “pre-loading” hereafter) before light-exposure. At 16 hrs post-illumination, promastigotes treated with Pc 3.5 became more granular, whereas those with csPcs 14/15 lost motility, but remained morphologically intact (not shown). By MTT assays, both parasites and MCs were found, as expected, sensitive to the photolytic activities of the 3 csPcs, but the kinetics of their susceptibility varied greatly with the Pc concentration (**Figure 2 C–E**). Viability of the MCs decreased with increasing csPc concentrations (**Figure 2 C–E** **green triangle**), whereas that of both *Leishmania* stages remained unchanged initially until the csPc reached higher loading concentrations of 10–100 µM (**Figure 2 C–E** **, red & black squares**). At these concentrations, the 3 csPcs were 20–50 fold more photolytic to *Leishmania* than to the MCs. *Leishmania* treated with ≥10 µM csPcs and light-exposed failed to grow when inoculated into their culture medium for incubation for up to 7 days (not shown). MCs also behaved similarly but only when treated with 100 µM csPc 3.5. The necessity of prolonged cell-csPc incubation for manifestation of the phototoxic phenotypes suggests that cellular uptake of the csPcs is a prerequisite for their effectiveness. This was shown clearly by fluorescence microscopy of *Leishmania* promastigotes (**Figure 3 A–C**) and axenic amastigotes (**Figure 3 A′–C′**) pre-loaded with the 3 representative csPcs. Cells exposed to csPc3.5 produced more intracellular fluorescence than those to csPcs 14/15, as noted by both fluorescence microscopy and flow cytometry (not shown). Overall, the same csPcs are photolytically more effective against both stages of *Leishmania* than their host cells under certain conditions, while none of the Pcs examined is cytotoxic without illumination.

### Photolytic activities of the effective csPcs vary with their specificity of targeting to different cell organelles in both *Leishmania* and macrophages

**Figure 4:**
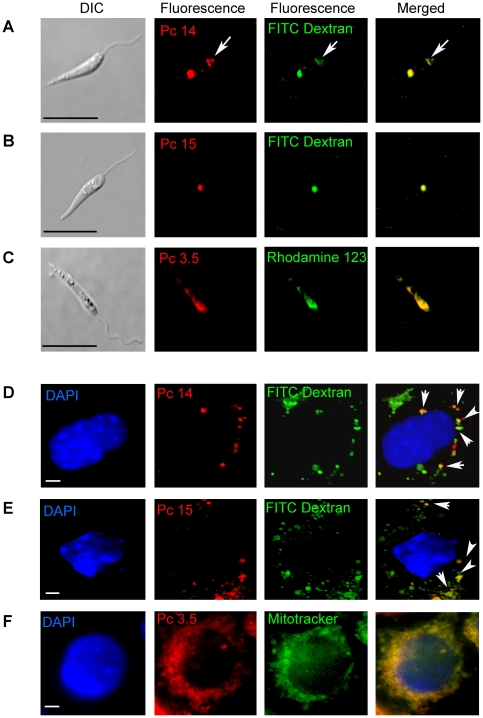
Localization of csPcs to cell organelles in *Leishmania* and macrophages. [**A–C**] *Leishmania* and [**D–F**] J774 MCs preloaded with 10 µM csPcs for 16 hrs. **A–F**-2^nd^ column, csPc-positive fluorescence; **A–B and D–E**-3rd column, endocytic vesicles labeled with FITC-dextran; **C**-3rd column, *Leishmania* mitochondria with rhodamine 123; **F**-3rd column, MC mitochondria with mitotracker green. MC nuclei DAPI-stained blue in D–F. A–B and D–E “merged” shows csPc 14 and 15 colocalization with endocytic vesicles. C and F “merged” show csPc 3.5 & mitochondria co-localization. Scale bar = 10 µm.

Fluorescence microscopy of csPc-exposed *Leishmania* and MCs showed that anilinium csPc 3.5 and pyridyloxy csPcs 14/15 co-localized with mitochondrial and endocytic markers, respectively (**Figure 4**). By the same approach, we observed that csPcs 11–13 and csPcs 3.6/3.7 were also endocytic, while csPcs 3.2/3.4 mitochondrial; and that Pc 10 was cytosolic in MCs, but undetectable in *Leishmania* (not shown). DIC imaging of *Leishmania* (**Figure 4 [A–C]**) and 4′, 6-diamidino-2-phenylindole (DAPI)-staining of MC's nuclei (Figure 4 **[D–F]**) showed their cellular integrity and provided reference for orientation of their cell organelles. csPcs 14/15 co-localized with the endocytic marker, i. e. Fluorescein isothiocyanate labeled dextran (FITC-dextran), in both *Leishmania* and MCs (**Figure 4 [A–B] and [D–E]** **merged panels**). *Leishmania* endocytic vesicles are known to aggregate into a single multi-vesicular body, which was rendered visible apparently by the accumulation of FITC-dextran and/or csPcs to a sufficient level in this site (**Figure 4 [A–B] and [D–E]** **2^nd^ and 3^rd^ panels**). In *Leishmania*, both csPc fluorescence (**Figure 4 A** **, 2^nd^ panels**, white arrow) and FITC-dextran fluorescence (**Figure 4 A** **3^rd^ panels**, white arrow) were also detected in the flagellar reservoirs where extracellular molecules are taken up via endocytically active lining membrane. In MCs, csPc 14/15 fluorescence (**Figure 4 [D–E]** **2^nd^ panels**) and FITC-dextran fluorescence (**Figure 4 [D–E]** **3^rd^ panels**) were seen in vesicles scattered in the cytoplasm. Many csPc-positive vesicles overlapped with those containing FITC dextran (**Figure 4 [D–E]** **2^nd^, 3^rd^ & merged panels, white arrows**), indicating that csPc15 was also taken up by MCs endocytically. CsPc 3.5 fluorescence was seen to overlap with the rhodamine 123 mitochondrial marker of *Leishmania* (**Figure 4 C**) and with the staining pattern of mitotracker green in MCs (**Figure 4 F**). The cellular targeting specificity of these different csPcs is underscored by a lack of co-localization of Pc15 with mitotracker, and of Pc 3.5 with dextran-FITC in both *Leishmania* (**Figure S1, A–B**) and MCs (**Figure S1, C–D**).

Notably, the cellular targeting of these csPcs is correlated with their photolytic potency: the mitochondrial csPc 3.5 is more photolytic to *Leishmania* (Cf Figure 2 A and B) and produced much more ROS in light-exposed lysates (data not shown) than the endocytic csPcs 14/15.

### Phagolysosomal *Leishmania* were differentially sensitized for photolysis by treating infected macrophages with endocytic csPc, but not with mitochondrial csPc

**Figure 5:**
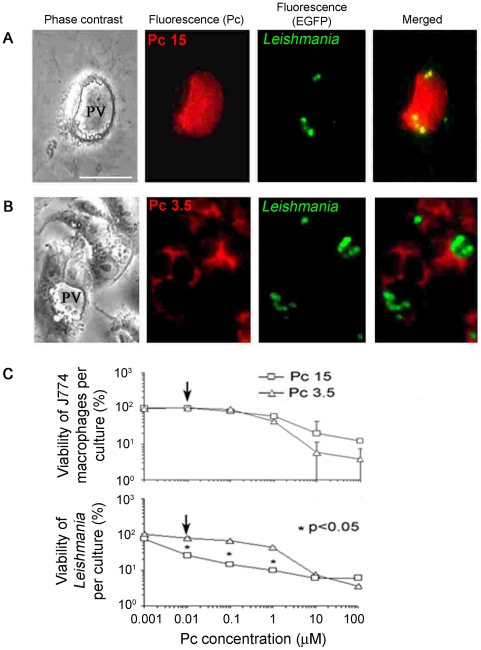
Exposure of infected macrophages to endocytic and mitochondrial csPcs differentially sensitize phagolysosomal GFP-*Leishmania* for photolysis. [**A–B**] **csPcs localization by fluorescence microscopy.** **PV** (**Phase contrast**), Large *Leishmania*-containing phagolysosomes after infection of MCs with GFP-transfectants. Exposure of these cells to csPcs 15 and 3.5 (10 µM) resulted in their fluorescence at different sites: **[A](Pc)**, csPc 15 in the PV; **[A](EGFP)**, GFP-fluorescent *Leishmania* in PV; **[A]Merged**, Co-localization of the two in the same PV; **[B](Pc)**, csPc 3.5 in the cytoplasm outside of PV; **[B](EGFP)**, GFP-*Leishmania* in PV; **[B] Merged**, No co-localization of the two. Scale bar: 100 µm. [**C**] **Viability of infected macrophages and their intracellular** ***Leishmania*** **.** Adherent *Leishmania*-infected MCs as described above were exposed to increasing concentration of Pc 3.5 and Pc 15, washed and light-exposed. **Upper panel**, MTT assays for MC viability after 16 hrs at 35°C. **Lower panel**, MTT assays for viability of intracellular *Leishmania* released from PT-treated MCs and cultured for 7 days. □ & **Δ**, Samples exposed to Pc 15 and Pc 3.5, respectively. **Arrows**, csPc 15 versus csPc 3.5 at 0.01 µM for photolysis of intracellular *Leishmania* without affecting host cell viability. *, p<0.05.

*L. amazonensis* infection is known to distend the phagolysosomes of MCs into large parasitophorous vacuoles (PV) (**Figure 5 [A]–[B]** **Phase contrast, PV**), rendering the parasites therein easily visible, especially when using *Leishmania* transfectants expressing green fluorescent protein (GFP) (**Fluorescence-EGFP**). Exposure of the infected MCs to the endocytic csPc15 led to its accumulation in these PV (**Figure 5 [A]** **Fluorescence Pc**) and thus co-localization with the GFP-*Leishmania* (**Figure 5 [A]** **Merged**). Co-existence of the csPc and the intra-PV *Leishmania* is suggested by the mergence of GFP-csPc fluorescence (yellow) in most of them. In contrast, exposure of similarly infected MCs to the mitochondrial csPc 3.5 resulted in its cytoplasmic or mitochondrial fluorescence (**Figure 5 [B]** **Fluorescence Pc**), but no co-localization with GFP *Leishmania* in the PV (**Figure 5 [B]** **Fluorescence GFP and Merged**).

The anti-*Leishmania* PT potential of endocytic csPcs was shown by illumination of the infected MCs after treatment with csPc15 in comparison to csPc 3.5 (**Figure 5 C**). By MTT assays, the viability of the host cells was found to decrease dose-dependently following similar kinetics in the presence of both csPcs (**Figure 5 [C]** **Upper panel**). Fluorescence microscopy of these cells for the intra-PV GFP-*Leishmania* initially revealed that the intensity of their GFP fluorescence diminished after treatment with csPc15, but not with csPc 3.5 (not shown). This difference was shown quantitatively by MTT assays of the surviving parasites, which were recovered from treated cultures for growth as promastigotes *in vitro* (**Figure 5 [C]** **Lower panel**). Infected cultures treated with both csPcs at higher concentrations of 10–100 µM yielded few viable MCs and no viable *Leishmania*. At the lower concentration range of 0.001 to 1 µM, the viable *Leishmania* recovered per culture of infected MCs was 3–4 fold less when treated with csPc 15 than with csPc 3.5 (**Figure 5 [C]** **square vs triangle**). Significantly, the photolytic suppression of viable parasites to this lower level was accompanied by no loss of host cell viability at 0.01 µM of csPc 15 (**Figure 5 [C]** **Arrows**).

The intracellular targeting of endocytic csPc 15 and mitochondrial csPc 3.5 correlates well with their differential activities seen against the phagolysosomal *Leishmania* in infected cells. Nevertheless, the margin of parasite versus host selectivity for the photolytically effective concentrations of csPc 15 is small. This limitation is not unexpected, considering the presence of endocytic csPc 15 not only in the phagolysosomes but also in some endosomes, which may be less ROS-resistant.

### CsPc pre-loaded *Leishmania* were as infective to host cells as the untreated *Leishmania* and were selectively photolysed substantially, leaving host cells unaffected

**Figure 6:**
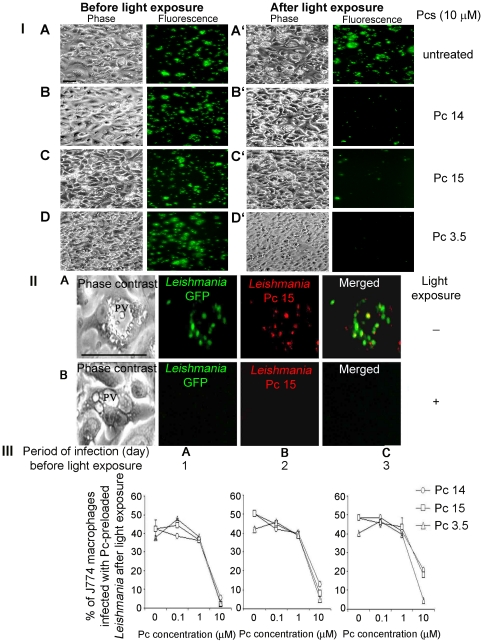
Infection of macrophages with Pc-preloaded *Leishmania* and selective photolysis of the latter after illumination. [**I**] **Fluorescence microscopy of infected cells showing light-mediated clearance of GFP** ***Leishmania*** **.** MCs infected with GFP-*Leishmania* [**A**] and those csPc-preloaded [**B–D**] were light-exposed. Phase contrast and fluorescence microscopy of cells for GFP before [**A–D**] and 1 day after light exposure [**A′–D′**]. **Note:** The integrity of macrophage monolayers and the *Leishmania* green fluorescence clearance, except in the control (**untreated**). Scale bar: 100 µm. [**II**] **Fluorescence microscopy of csPc-/GFP-positive** ***Leishmania*** **in PV and their clearance by light exposure.** Scale bar: 100 µm. **Upper panel:** PV containing *Leishmania*, which fluoresce green (GFP), red (csPc) and yellow (merged) before light-exposure. **Lower panel:** An empty PV cleared of all fluorescent *Leishmania* after light-exposure. [**III**] **Flow cytometry of GFP-** ***Leishmania*** **-infected MCs for GFP fluorescence showing csPc concentration-dependent photo-clearance of the infection:** The same culture sets as [I] infected with GFP-*Leishmania*, but loaded with increasing concentrations of the 3 csPcs indicated. Cells were collected daily for light-exposure in 3 consecutive days (**1–3**). [**A–C**] Flow-cytometry of cells from days 1, 2 and 3 for GFP fluorescence intensity as a measure for the infection.

The parasite versus host specificity for photolysis was enhanced significantly when *Leishmania* pre-loaded with csPcs were used to infect host cells. For this study, MCs and DCs were infected with GFP-transfectants to simplify the evaluation of infection by fluorescence. Pre-loading of these transfectants with csPcs 3.5, 14 and 15 (10 µM) in the dark was found to produce no deleterious effects, leaving them fully motile and viable. These csPc-loaded *Leishmania* were as infective as untreated GFP-transfectants, producing similar intensities of intracellular GFP fluorescence 2 days after infection of MCs (**Figure 6 [I] A–D** **, Before light exposure: Phase and Fluorescence of untreated, csPcs 14, 15 & 3.5**) and DCs (**Figure S2 A–B, Before light exposure: Phase and Fluorescence of untreated & csPc 3.5**). At higher magnification (**Figure 6 [II]A**), the PVs of infected MCs were clearly seen to contain *Leishmania* fluorescent in green due to GFP, in red due to csPc and yellow for the presence of both. (Note: Population heterogeneity of both GFP- and csPc-*Leishmania* in fluorescence intensity is expected, precluding the visualization of combined fluorescence in all individual cells). Light exposure of the infected cells harboring csPc-loaded *Leishmania*, but not untreated *Leishmania*, substantially cleared the infection, as indicated by the disappearance of the cellular GFP fluorescence from both MCs (**Figure 6[I] A′–D′** **After light exposure: Phase contrast and fluorescence of untreated versus csPcs 14, 15 & 3.5**) and DCs (**Figure S2 A′–B′, After light exposure: Phase and fluorescence of untreated versus csPcs 3.5**). Persistence of GFP fluorescence in csPc-untreated, but light-exposed controls indicates that it is not sensitive to photo-bleaching under the conditions of illumination used. The loss of GFP fluorescence is thus accounted for by the degradation of GFP, as the GFP-*Leishmania* were photolysed progressively in the PV, which became smaller and devoid of visible *Leishmania* (**Figure 6 [II] B**). Significantly, the host cells remained undisturbed, as indicated by their persistence as monolayers of confluent adherent cells (**Figure 6 [I] A–D versus A′–D′** **; Figure S2 A–B versus A′–B′, Phase contrast**) and by their comparable MTT reducing activities (not shown) before and after light exposure.

The observation was further verified under optimal conditions by infecting host cells with GFP-*Leishmania*, which were pre-loaded with decreasing concentrations of csPcs. The selectivity and efficacy of the photolytic clearance of csPc-loaded *Leishmania* from these infected cells was clearly shown quantitatively by flow cytometry for GFP fluorescence (**Figure 6 [III]**). In all cases, the % of cells with GFP-fluorescence or *Leishmania* infection decreased after light exposure proportionally with increasing csPc loading concentrations; the most striking decrease being from 1 to 10 µM (**Figure 6 [III]** **and figure S2 [II]**). At the highest csPc loading concentration of 10 µM, photolytic clearance of the infection reached almost 100% when assessed 1 day after illumination, but was reduced thereafter with additional days of incubation in the dark before illumination (**Figure 6 [III]** **A–C, 1–3**).

The results obtained indicate that *Leishmania* pre-loaded with csPcs retained their innate ability of homing to phagolysosomes of MCs and DCs. The PS is thus delivered specifically by *Leishmania* to this ROS-resistant site, accounting for the specificity and efficiency of leishmanolysis.

### Uptake of csPc pre-sensitized and pre-illuminated *Leishmania*, and their intracellular photo-clearance from macrophages

**Figure 7:**
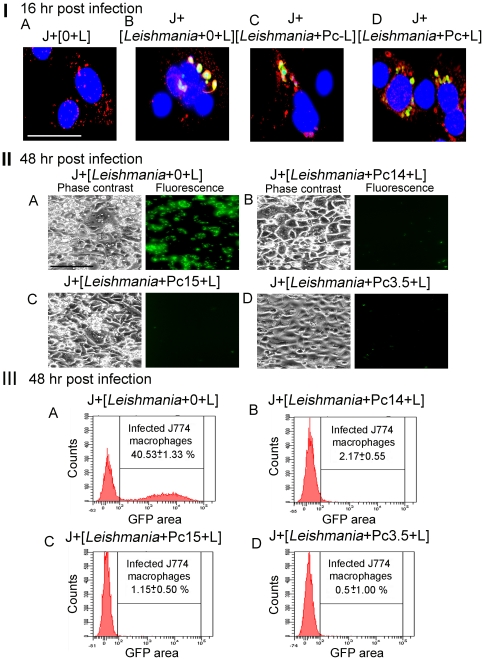
Infection of J774 macrophages with csPc-preloaded/pre-illuminated GFP-*Leishmania*, and their photolytic clearance. [**I**] **Endocytosis of Pc-preloaded/pre-illuminated GFP-** ***Leishmania*** **by J774 macrophages.** [**A**] MCs light-exposed (J[+0+L]); [**B**] As [A], but pre-infected with GFP-*Leishmania* (J+[*Leishmania*+0+L]); [**C**] As [B], but infected with Pc 14-preloaded *Leishmania* without light-exposure; ([*Leishmania*+Pc−L]); and [D] As [C], but infected with Pc-preloaded/pre-illuminated GFP-*Leishmania* (J+[*Leishmania*+Pc+L]). Immunofluorescence microscopy of all cells 16 hr post-infection for EEA-1 endosome marker. **Green**, GFP-*Leishmania*; **Blue**, DAPI-stained MC nuclei; **Red**, EEA1-positive endosomes. **Note:** co-localization of *Leishmania* GFP with endosome marker. Scale bar: 100 µm. [**II**] **Photolytic clearance of Pc-preloaded/pre-illuminated GFP-** ***Leishmania*** **from infected cells.** MCs were infected with GFP-*Leishmania* **([A])**, and those preloaded with the 3 csPcs as indicated ([**B–D**]) and light-exposed. Phase contrast and fluorescence microscopy of infection after 2 days. **Note**: Clearance of GFP from all doubly treated cultures without affecting the appearance of host cell monolayers. Scale bar: 300 µm. [**III**] **Flow-cytometric quantitation of GFP fluorescence** of the same samples used for [II], showing a ∼40% infection rate in the control (**[A] GFP**) reduced to negligible levels in the doubly treated groups (**[B–D] GFP**).

CsPc-loaded promastigotes were noted to remain structurally intact long after light exposure. Although these doubly treated GFP-*Leishmania* were unable to grow and perished eventually (see preceding section), they were found to infect host cells as well as those treated with csPc alone or light alone (not shown). Endocytosis of all these GFP-*Leishmania* by MCs was verified by immuno-staining their endosomes for Early Endosome Antigen 1 protein (EEA1). This marker labeled the endosomes of uninfected cells as red fluorescent cytoplasmic vesicles (**Figure 7[I]A**) and co-localized with fluorescent GFP-*Leishmania* in the phagosomes of the MCs, regardless of whether *Leishmania* were csPc-preloaded, pre-illuminated, treated with both or untreated (**Figure 7 [I] B–D J+[** ***Leishmania*** **+0+L, +Pc−L, and +Pc+L]**). After incubation for 2 days, the MCs remained infected by the control parasites (**Figure 7** **[II] A and [III] A**), but were cleared of the doubly treated *Leishmania*, irrespective of their pre-loading with csPcs 3.5, 14 or 15 before pre-illumination (**Figure 7 [II] B–D**). Quantitative flow cytometry of these cells further revealed that the GFP-positive populations were significantly reduced by ∼20, ∼35 and ∼80 fold for csPcs 14, 15 and 3.5, respectively (**Figure 7 [III] B–D**). The clearance of the infection from these cultures appeared to be complete, since csPc-loaded/pre-illuminated *Leishmania* failed to grow. Infection of DCs with csPc-pre-loaded and pre-illuminated *Leishmania* produced a similar outcome (not shown).

The results obtained indicate that immediately after csPc-loading/illumination *Leishmania* remain infective, but are substantially cleared rapidly and selectively.

### Photolytic delivery of ovalbumin by *Leishmania* to DCs presents an MHC Class I-restricted ovalbumin peptide that activates its specific CD8+ T cell line *in vitro*

**Figure 8:**
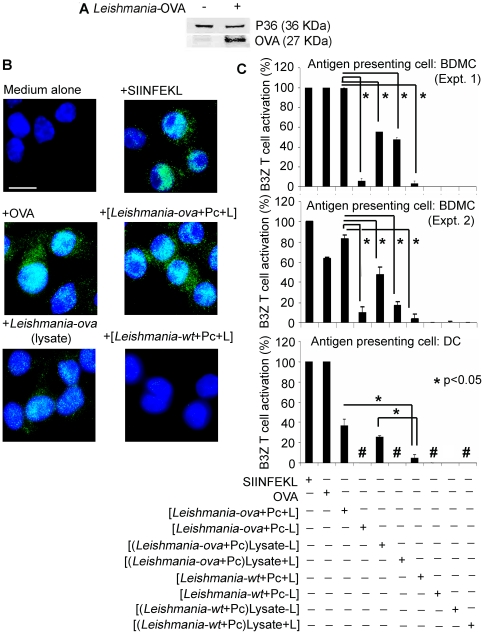
*Leishmania* expression of ovalbumin and its photolytic delivery to APCs for antigen presentation *in vitro*. [**A**] **OVA expression by** ***Leishmania*** **transfectants.** Western blot of wild type and transfectants (**ova**) showing the presence of the *Leishmania* P36 in both, but only OVA in the latter. [**B**] **Immunodetection of the OVA-SIINFEKL/MHC Class I complex co-presentation in infected DC.** OVA-expressing and WT *Leishmania* preloaded with 10 µM csPc 14 and light-exposed for 45 mins were prepared. DCs were exposed at 35°C for 16 hrs to the following conditions: **Negative controls**, **Medium alone** and Pc-/light-exposed WT *Leishmania* (**+** ***Leishmania*** **-wt+Pc+L**); **Positive controls**, 100 pM SIINFEKL peptides (**+SIINFEKL**) and 5 mg/ml chemically pure native ovalbumin (**+OVA**); and **experimental group**, Pc- and light-exposed ova-transfectants (+[***Leishmania*** **-ova+Pc+L**]) and their lysates without light exposure (+[***Leishmania*** **-ova (lysate)**). DC to *Leishmania* ratio used = 1∶100. Treated cells were reacted with the monoclonal specific for SIINFEKL/MHC class I molecule complex for immunofluorescence microscopy. **Note**: Fine green granular products = positive reactions; **Blue**, DAPI-stained DC nuclei. Scale bar: 50 µm. [**C**] **Activation of OVA-specific CD8+ T cells by BDMCs and DCs with OVA-expressing** ***Leishmania*** **:** Positive and negative controls are described in legends below the graph. Infected DCs and BDMCs were co-cultured with the OVA MHC class I epitope (SIINFEKL)-specific CD8^+^ T cell hybridoma (B3Z). LacZ reporter gene activity measured for OVA epitope-specific B3Z T cell activation, as described. p values<0.05, as calculated by student's t-test. Data are presented from 2 independent experiments using BDMC and 1 representative experiment using DC as the APCs. **#**, not done.

*Leishmania* transfected with the cDNA encoding a truncated OVA was used to serve as a carrier for this xenogenic, albeit endogenously expressed, T cell model antigen of 27 kDa (aa 140–386) (OVA) (**Figure 8[A]** **, OVA**). The transfectants, which were csPc 15 pre-loaded and pre-illuminated, remained infective to DCs (not shown) under the experimental conditions used for the similarly pre-treated wild-type or GFP-*Leishmania* (Figure 7). OVA delivered in this way to DC was apparently processed correctly by these antigen-presenting cells (APC) to present the known MHC Class I-specific SIINFEKL epitope. This is indicated by the positive reaction of this MHC-epitope complex with a monoclonal antibody 25-D1.16, which is known to have this specificity [25] (**Figure 8[B]**). The positive immuno-reaction products, in green or pale blue when overlapped over DAPI-stained nuclei, were present in DCs infected with these photo-inactivated transfectants (**Figure 8[B]** **+[Leish-ova+Pc+L]**) at levels as in those exposed to all the SIINFEKL-positive controls (+SIINFEKL peptides, +OVA, +Leish-ova lysates), but not in the negative controls (Medium alone, +[Leish-wt+Pc+L]). In addition, in 3 independent experiments (Figure 8[C]), both DCs and bone marrow-derived MCs (BDMC) infected with the photo-inactivated transfectants [Leish-ova+Pc+L] were found to activate CD8+ Ova specific T cells (B3Z T cells), which are known to react specifically with the MHC Class I/SIINFEKL epitope complex, resulting in the expression of Lac Z as the readouts [26] (**Figure 8[C]**). Based on this assay under the experimental conditions used, B3Z T cells were activated by co-cultivation with DC/BDMC+[Leish-ova+Pc+L] or +Leish-ova lysates to a significant level that was ∼40% of those with DC+ SIINFEKL peptides or +OVA, and virtually identical to those of BDMC+ SIINFEKL. csPc-loaded *Leishmania* without illumination [Leish-ova+Pc−L] remained infective and viable in BDMC; activation of B3Z T cells by these infected BDMC was of the background level, e. g. Leish-WT+Pc±L. All other negative controls produced little or no activation (**Figure 8[C]** see legends at the bottom of the bar graph).

The results thus indicate that foreign antigens can be expressed by *Leishmania* for csPc-mediated photolytic delivery to APC for presentation to activate epitope-specific T-cells *in vitro*.

## Discussion

This is the first report showing that both stages of *Leishmania* are intrinsically susceptible to the photolytic activities of soluble and cationic Zn−/Si-Pcs (csPcs) examined (Figure 1 and 2 [A–B]). Since the axenic amastigotes are closer to the disease-causing stage of *Leishmania*, their intrinsic and irrevocable susceptibility to csPc-mediated cell death is especially relevant in considering csPcs as agents for therapeutic PT against cutaneous leishmaniasis.

Photolytic activity of the csPcs requires their uptake by cells (Figure 3), consistent with the outcome of our observations with endogenously generated URO [24]. Additions of anilinium or pyridyloxy groups, axial ligands and/or PEGylation to the core structure of the Pc (Figure 1) apparently facilitate the cellular uptake of these csPcs. These modifications increase their cationicity for enhancing interaction with the negatively charged cell surface, and their solubility for increased bioavailability [27] and decreased self-quenching [28]. Subcellular localization of the representative csPcs (Figure 4) suggests that the mechanisms of their cellular uptake follow at least 2 different pathways, common to both *Leishmania* and macrophages: endocytosis for pyridyloxy csPcs, e. g. 14/15, and plasma and mitochondrial membrane transport of di-anilinium csPcs, i. e. Pc 3.5. It is not known whether the mitochondrial import of this csPc utilizes a specific transporter, as reported for a different Pc series, e. g. Si-Pc4 [29]. Further study of the structure-function relationships of these and other csPcs are needed to elucidate the precise mechanisms of their cellular uptake and trafficking.

Our results together with those from previous work show that the subcellular targeting differences of the PS figure significantly in the photolytic phenotype observed. The subcellular targeting specificity of the effective csPcs presented here differs from that, which we reported previously, for endogenously induced URO [24] and exogenously applied AlPhCl [7]. The csPcs accumulate gradually in *Leishmania*, akin in timeframe to the neogenesis of URO in porphyric mutants [23], [30], but in different sites, resulting in the manifestation of very different phototoxic phenotypes. Flagellar motility was rapidly paralyzed by light exposure of the uroporphyric mutants when URO began to emerge in their cytosol [24], [30], but not when *Leishmania* was pre-loaded with csPcs in their endosome/phagolysosomes or mitochondria. These PS-sensitized *Leishmania* do not lose their viability immediately after illumination in sharp contrast to the outcome of those treated with membrane-associated AlPhCl [7]. The cellular targeting specificity of these and other csPcs warrants further study to understand their mechanisms in relation to their observed differences in photodynamic properties.

In the present study, evidence is also presented for the first time that the endocytic PS, like csPcs 14/15, are potentially useful for therapeutic PT against phagolysosomal pathogens, e. g. *Leishmania* spp. The specificity of these PS for targeting phagolysosomal *Leishmania* accounts more for their effectiveness than their intrinsic photolytic activities, as the mitochondrial csPcs are more photolytic to promastigotes, but less leishmanolytic against those in infected cells than the endocytic csPcs 14/15 (Figure 5A–B). The endocytic csPcs are expected to be effective for PT *in vivo* by just clearing the infection of some infected MCs so that they, once free from *Leishmania*-mediated immunosuppression, are able to initiate effective immunity to clear the remaining infection. This scenario is consistent with some measure of success of PT using other PS reported against clinical cutaneous leishmaniasis [6], [8]. The use of endocytic csPcs is expected to significantly enhance both pharmacological effectiveness of PT as well as the post-therapeutic immune clearance of *Leishmania* infection. For such applications, csPcs may be further modified for lysosomal activation [31] to increase the margin of parasite versus host selectivity.

Our *in vitro* data presented support our proposal that the PS-loaded *Leishmania* are potentially useful carriers to deliver drugs/vaccines to the appropriate site for pharmacological/immunological activation [23]. *Leishmania* pre-loaded with csPcs provide an additional carrier inducible for destruction (Figure 6) as alternatives to the uroporphyrinogenic mutants [23]. The csPcs appear “locked up” in the cell organelles more tightly than membrane-associated AlPhCl [7], thereby avoiding “leaching out” to sensitize host cells for photolysis, as found with the latter. Pre-illumination of these csPc-loaded *Leishmania* eliminates their ability to grow, thereby increasing the safety margin of their future applications (Figure 7). Also, the clearance of *Leishmania* from infected cells requires no additional illumination, thereby simplifying the experimental steps. While persistence of a few *Leishmania* below detection can never be ruled out, they are expected to succumb to post-PT immune clearance under *in vivo* conditions, as noted previously [22].

Evidence is further provided for the first time that specific antigen can be expressed by *Leishmania* for photolytic delivery after PS-loading to DC or BDMC to elicit a T cell response, supporting our proposal for their utility as a vaccine carrier in immuno-prophylaxis and –therapy. Transfection of *Leishmania* to express OVA makes it possible to photolytically deliver it as a surrogate vaccine for *in vitro* evaluation of T cell specific immune response (Figure 8). Significantly, csPc-loaded transfectants are able to deliver OVA to DCs and MCs for appropriate processing. Pre-illumination of csPc 14/15-loaded transfectants gave the most consistent results, suggesting that the photolytic environment of the PT preserve not only the carrier capacity of the transfectants but also the antigenicty of OVA epitopes in these cells. Delivery of OVA by photo-inactivated *Leishmania* to BDMC for this activity is especially impressive, as it is higher even than that produced by the lysates of these *Leishmania* that were supplied to APC in equivalent amounts (Figure 8C). While DCs and MCs are susceptible to the infection by the csPc-loaded transfectants and illumination of these infected cells cleared the infection (Figure 6), delivery of OVA in this way for antigen presentation produced less consistent results (not shown). Work is still on-going to optimize the experimental conditions. OVA SIINFEKL-MHC Class I co-presented by the infected DCs and BDMCs is functionally active, since such APCs are capable of activating SIINFEKL-specific CD8+ T cells. Work is underway to evaluate the lysosomal delivery of OVA for presentation of different OVA epitopes to specific CD4+ T cells. Completion of the work with this and other defined antigens is expected to provide the necessary foundation for future evaluation of vaccine candidates photolytically delivered by *Leishmania* against other diseases.

## Materials and Methods

### Phthalocyanines

Synthesis of pyridyloxy Pcs and their photophysical and photochemical properties have been reported [32]. The synthesis of anilinium Pcs used here will be published by Maria da Graca H. Vicente in details separately elsewhere. **Figure 1** shows the structures of anilinium Pcs (Pc 1–3.7) and pyridyloxy Pcs (Pc 10–15) examined in the present study. All Pcs were dissolved in dimethyl sulfoxide (DMSO) (Sigma) to 100 mM. The stock solutions were used immediately or stored in the dark at −20°C.

**Cells:** Used in this study were wild-type clone 12-1 of *Leishmania amazonensis* (RAT/BA/74/LV78) and its GFP transfectants [7], mouse macrophage cell line J774A1 (MC) [7], mouse bone marrow derived macrophages (BDMC), mouse dendritic cells of the DC2.4 line (DC) [33] and the B3Z T cell hybridoma [26]. *Leishmania* promastigotes, axenic amastigotes and J774A1 macrophages were grown as described [7]. BDMCs were differentiated from bone marrow cells of 129/C57BL6 mice and maintained in DMEM containing macrophage colony stimulating factor [34]. DC2.4 and B3Z T cell lines were grown in supplemented RPMI 1640 [26], [33]. *Leishmania* transfectants were grown for 1-cycle in drug-free medium and washed by centrifugation up to 3-times before use.

### OVA transfection/expression

Promastigotes were transfected by electroporation [23] with pX63*hyg*-*ova*, consisting of a truncated ovalbumin (OVA, aa 140–386) [35] cloned into the *Bgl II* expression site of pX63*hyg* [24]. Stable transfectants were selected and grown at 500 µg/ml of hygromycin [24]. OVA expression in the transfectants was assessed by Western blotting using anti-OVA rabbit antisera (Millipore, dilution: 1∶ 1000) and donkey anti-rabbit IgG labeled with fluorophore CW800 (Licor, dilution: 1∶ 20000). Blots were scanned for reaction products in an Odyssey infrared scanner (Licor). *Leishmania* constitutively expressed protein of 36 kDa (p36) were included as the loading control [36].

### *Leishmania* infection of host cells

MCs or DCs were mixed with *Leishmania* at a parasite-to-host cell ratio of 10∶1, i. e. 5×10^6^ *Leishmania*/5×10^5^ host cells/ml. Infection was initiated by plating the mixtures under the following conditions: [1] ∼0.5 ml/well in 24 well tissue culture plates for most studies; [2] 0.2 ml/well in 8 chamber microscopic slides for immunofluorescence microscopy. Infected cultures were incubated at 35°C, subjected to medium renewal, if necessary, and washed before use.

### *In vitro* photodynamic therapy

Late log-phase promastigotes/GFP transfectants and axenic amastigotes were treated with Pcs each in 10× serial dilutions (100 µM being the highest) at a cell density of 10^8^ cells/ml in Hank's Balanced Salt Solution plus 0.01% bovine serum albumin (HBSS-BSA) at pH 7.4 and pH 5.4, respectively [7], [30]. Promastigotes and axenic amastigotes so treated were incubated in the dark at 25 and 33°C, respectively.

*Leishmania*-infected (for 2–3 days) and non-infected cells at ∼10^6^ cells/ml were treated similarly with Pcs, but in their specific culture conditions. Negative controls included both *Leishmania* stages and infected/non-infected host cells, which were treated with the solvent of Pcs at the highest concentration used, i. e. 0.1% DMSO. DMSO at this concentration was not cytotoxic [7].

All Pc-treated cells were exposed to light with or without removing the Pcs from the incubation milieu, *Leishmania* cells were referred to as “pre-loaded” in the former case, i. e. 3× centrifugations of cells in HBSS each at 4°C for 5 min at 3,500 g. Host cell monolayers were 3× washed with the buffer. *Leishmania* were plated at 2×10^7^ cells/0.2 ml/well and host cells at 0.25–0.5×10^6^ cells/0.5 ml/well in 96-well and 24-well tissue culture plates, respectively. Illumination referred to as “light-exposure” was optimized as follows. The plated cells were placed at a distance of ∼3 cm from the light source at the bottom for illumination over a red filter (wavelengths >650 nm; part no. 650021; Smith-Victor Co., Bartlett, IL) under a constant temperature of ∼25°C. The light source was a light box, consisting of 2 white fluorescent tubes (15 watts each, General Electric; part no. F15T8CW) and a light diffuser on top. A L1-250A light meter (LI-COR) was used to read the irradiance, producing a value of 0.55 mW/cm^2^ that gave a fluence of 2.0 J/cm^2^ after exposure for the duration of 1 hr [7].

### Cell viability assays

Cells were assessed for their viability by microscopy, MTT reducing activities [24] and growth of the survivors [7]. For intracellular amastigotes, infected MCs were stripped from tissue culture plates by repeated flushing of individual wells with a Pasteur pipette. The cells suspensions were then vortexed vigorously to break infected macrophages for releasing intracellular amastigotes. Lysates in equal aliquots from different preparations were each incubated under promastigote culture conditions. After ∼7 days of growth, parasites were assessed for viability based on their MTT reducing activities.

### Fluorescence/immunofluorescence microscopy

Nikon Eclipse 80i and TE2000-S microscopes equipped with CCD cameras and Metamorphosis (version 6.1) software were used [24]. At least 50 individual cells were examined for each experimental and control set using specific filter sets (listed at the end).

**[1] Phthalocyanine subcellular localization:** Cells “pre-loaded” with 10 µM csPcs for 16 hrs were examined. **[2] Co-localization of csPcs and cellular organelle markers:** The following fluorescent markers were used: rhodamine 123 (0.2 mM) for *Leishmania* mitochondria, dextran-FITC (molecular weight of 10,000) (500 µg/ml) for *Leishmania* endosomes [24], mitotracker green FM (Invitrogen) for MC mitochondria and dextran-FITC (molecular weight of 40,000, Invitrogen) for MC endosomes. **[3] Treatment of GFP-** ***Leishmania*** **-infected macrophages with different csPcs.** MCs were infected with GFP-*Leishmania* for 3 days in 24 well plates, washed and exposed to 10 µM Pcs in the dark for 16 hrs and then examined by using the FITC filter set. **[4] Uptake of csPc-loaded/light-exposed GFP-** ***Leishmania*** **into EEA1-positive endosomes of macrophages.** MCs were infected for ∼16 hrs with GFP transfectants preloaded with Pcs (10 µM) and light-exposed. Untreated *Leishmania* and uninfected MCs were included as controls. Normal donkey serum was used to block non-specific interactions and rat anti-mouse CD16/32 antisera (eBiosciences) for Fc receptors. Cells were fixed/permeabilized with Cytofix-cytoperm (BD biosciences) for reaction with goat anti-EEA1 antisera (sc-6414, Santa Cruz Biotech) [37] and donkey anti-goat IgG-alexa594 (Molecular probes). **[5] Immunodetection of H-2K^b^ OVA_(257–264)_ (SIINFEKL) complexes of ova transfectant-infected DCs.** DC2.4 dendritic cells (5×10^4^) were exposed for 24 hrs at 37°C, 5% CO_2_, in 200 ul of complete medium to the following materials: 100 pM SIINFEKL, 5 mg/ml OVA, freeze thawed lysates of *Leishmania* transfectants expressing OVA (5×10^6^ promastigotes), csPc preloaded/light-exposed OVA transfectants or control untransfected cells (5×10^6^ promastigotes) and medium alone. Exposed cells permeabilized as earlier described were treated at 4°C for 16 hrs with the monoclonal from the 25-D1.16 hybridoma culture supernatants followed by goat anti-mouse IgG-alexa488 (Molecular probes) (1∶ 1000 dilution) to assess the H-2K^b^ OVA_(257–264)_ (SIINFEKL) [31]. **Fluorescence microscopy filter sets** (Chroma Technology Co., Brattleboro, VT) were used for the fluorescence microscopy as follows: [1] D365/10X (365 nm exciter), 400DCLP (400 nm dichroic) and D460/50M (460 nm emitter) for DAPI; [II] HQ480/40 (480-nm exciter), Q505LP (505-nm dichroic), and HQ535/50 (535-nm emitter) for green fluorescent protein (GFP), dextran-fluorescein isothiocyanate (dextran-FITC), rhodamine 123, mitotracker green and alexa 488; [III] HQ545/30 (545-nm exciter), Q570LP (570-nm dichroic), and HQ620/60 (620-nm emitter) for alexa 594; and [IV] HQ620/60 (620-nm exciter), Q660LP (660-nm dichroic), and HQ700/80 (700-nm emitter) for phthalocyanines.

### Antigen presentation assay

H-2K^b^ positive DCs or BDMCs were used to present OVA in various forms (see details at the bottom of Figure 8C) to the B3Z T cells [26], which express a TCR that specifically recognizes the OVA_(257–264)_ epitope (SIINFEKL) in the context of MHC I H-2K^b^. OVA-primed DCs or BDMCs and B3Z T cells were incubated at 1∶1 ratio for 24 hrs at 37°C in 96 or 24 well plates. *β–gal* expressed by the *lacZ* reporter gene of B3Z T cells [26] in response to MHC I+ SIINFEKL and TCR complex formation were assessed by a *β–gal-luciferase* coupled assay system (BETA-GLO Promega) as luminescence using Synergy HT plate reader (BioTek). The assay was pre-calibrated for optimal response of the T cells to the lowest concentrations of purified OVA (5 mg/ml) (Millipore) and SIINFEKL (100 pM) (AnaSpec) [26], [33]. In each experiment, the values obtained from the experimental groups were normalized against those from the positive controls as 100%.

### Flow cytometry

Infection of MCs and DC with GFP- or csPc-fluorescent *Leishmania* was quantitatively assessed by flow cytometry [38] using a Becton Dickenson flow cytometer (LSRII) equipped with BD bioscience software FACS DIVA for data acquisition and analyses [24].

All experiments were repeated 2–3 times. The data presented represent the means ± standard errors of the values in duplicate or triplicate for each of the individual samples from representative experiments. Statistical analysis was done using the student *t-test*.

### Ethics Statement

Animals (129/C57BL6 mice) used in this study were maintained under strict accordance with the recommendations in the Guide for the Care and Use of Laboratory Animals of the National Institutes of Health. The protocol was approved by the IACUC at RFUMS (Protocol Number: 11-08). All animals were appropriately treated to minimize their undue discomfort and euthanized humanely under isoflurane anesthesia.

## Supporting Information

Figure S1 **Co-localization of csPcs to cell organelles in** ***Leishmania*** **and macrophages.** [**A–B**] *Leishmania* and [**C–D**] J774 MCs preloaded with 10 µM of different csPcs for 16 hrs. **A–B**-2^nd^ column and **C–D** 1^st^ column, csPc fluorescence; **A**-3^rd^ column and **C**-2^nd^ column, mitochondria labeled with mitotracker green; **B**-3^rd^ column and **D**-2^nd^ column endocytic vesicles labeled with FITC-dextran. **Note:** No significant co-localization of csPc 15 fluorescence with mitotracker, and csPc 3.5 with endocytic marker (Merged and enlarged columns A–D). This is further clearly shown by line scans for intensity along the “white line” in enlarged column A–D. Scale bar = 10 µm.
(DOC)

Figure S2 **Infection of DCs with csPc 3.5-loaded** ***Leishmania*** **and selective photolysis of the latter after illumination of infected cells.** [**A–B, A′–B′**] **Phase contrast and fluorescence microscopic images of adherent DC 2.4 cells showing clearance of GFP-** ***Leishmania*** **infection:** GFP transfected *Leishmania* (see green fluorescence) were loaded overnight with or without 10 µM csPc 3.5 and used to infect DC 2.4 cells. Infected monolayers were washed to remove non-attached extracellular parasites and light-exposed. Cells were examined by phase contrast and GFP fluorescence microscopy immediately before [**A–B**] and 1 day after light exposure [**A′–B′**]. **Note:** The integrity of the DCs and the substantial clearance of *Leishmania* green fluorescence from all cultures, except the control infected with *Leishmania* without Pc pre-loading (**untreated**). Scale bar = 100 µm. [**C**] **GFP flow cytometry of infected cells, showing substantial clearance of GFP** ***Leishmania*** **infection:** Similar culture sets as above were infected for 2 days with csPc-preloaded (10 µM Pc 3.5) or control *Leishmania*, as indicated. Cells were then light-exposed and detached with trypsin-EDTA (Invitrogen) 1 day after light exposure. Cells were assessed by flow-cytometry for GFP fluorescence as a measure of infection. **Note:** The significant loss of GFP fluorescence due to *Leishmania* photolysis in the DCs of the experimental group, but not of the controls.
(DOC)
