# Supplementary material for: Intracellular Targeting Specificity of Novel Phthalocyanines Assessed in a Host-Parasite Model for Developing Potential Photodynamic Medicine
Source: PLoS One. 2011 Jun 6;6(6):e20786. doi: 10.1371/journal.pone.0020786 (PMC3108980; doi:10.1371/journal.pone.0020786)
Supplement: Figure S1 — Co-localization of csPcs to cell organelles in Leishmania and macrophages. [A–B] Leishmania and [C–D] J774 MCs preloaded with 10 µM of different csPcs for 16 hrs. A–B-2nd column and C–D 1st column, csPc fluorescence; A-3rd column and C-2nd column, mitochondria labeled with mitotracker green; B-3rd column and D-2nd column endocytic vesicles labeled with FITC-dextran. Note: No significant co-localization of csPc 15 fluorescence with mitotracker, and csPc 3.5 with endocytic marker (Merged and enlarged columns A–D). This is further clearly shown by line scans for intensity along the “white line” in enlarged column A–D. Scale bar = 10 µm. (DOC) [file pone.0020786.s001.doc]

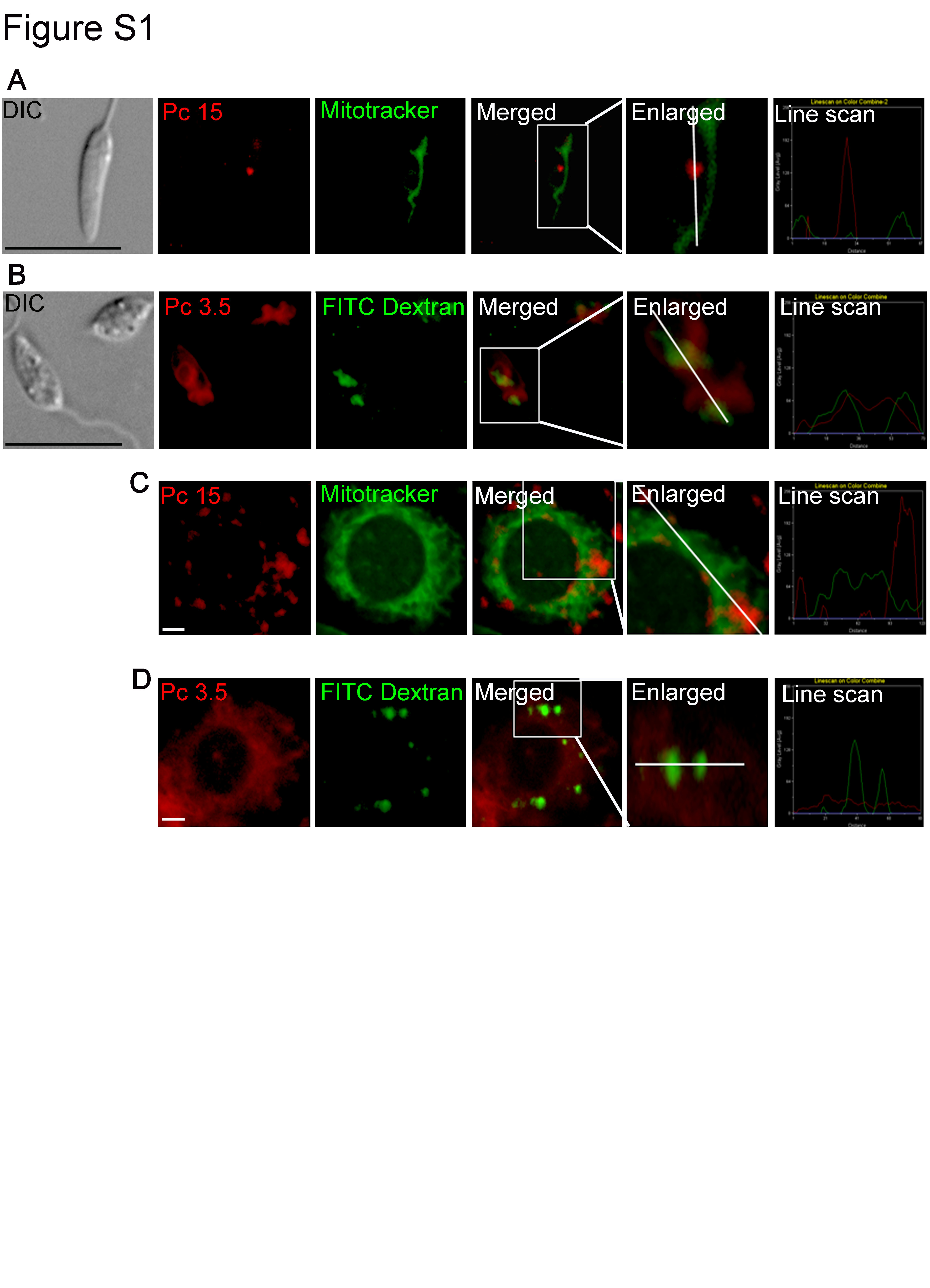


**Figure S1. Co-localization of csPcs to cell organelles in *Leishmania* and macrophages.**

**[A-B]** *Leishmania* and **[C-D]** J774 MCs preloaded with 10 µM of different csPcs for 16 hrs. **A-B**-2^nd^ column and **C-D** 1^st^ column, csPc fluorescence; **A-**3^rd^ column and **C**-2^nd^ column, mitochondria labeled with mitotracker green; **B**-3^rd^ column and **D**-2^nd^ column endocytic vesicles labeled with FITC-dextran. **Note:** No significant co-localization of csPc 15 fluorescence with mitotracker, and csPc 3.5 with endocytic marker (Merged and enlarged columns A-D). This is further clearly shown by line scans for intensity along the “white line” in enlarged column A-D. Scale bar = 10 µm.
