# Supplementary material for: Intracellular Targeting Specificity of Novel Phthalocyanines Assessed in a Host-Parasite Model for Developing Potential Photodynamic Medicine
Source: PLoS One. 2011 Jun 6;6(6):e20786. doi: 10.1371/journal.pone.0020786 (PMC3108980; doi:10.1371/journal.pone.0020786)
Supplement: Figure S2 — Infection of DCs with csPc 3.5-loaded Leishmania and selective photolysis of the latter after illumination of infected cells. [A–B, A′–B′] Phase contrast and fluorescence microscopic images of adherent DC 2.4 cells showing clearance of GFP- Leishmania infection: GFP transfected Leishmania (see green fluorescence) were loaded overnight with or without 10 µM csPc 3.5 and used to infect DC 2.4 cells. Infected monolayers were washed to remove non-attached extracellular parasites and light-exposed. Cells were examined by phase contrast and GFP fluorescence microscopy immediately before [A–B] and 1 day after light exposure [A′–B′]. Note: The integrity of the DCs and the substantial clearance of Leishmania green fluorescence from all cultures, except the control infected with Leishmania without Pc pre-loading (untreated). Scale bar = 100 µm. [C] GFP flow cytometry of infected cells, showing substantial clearance of GFP Leishmania infection: Similar culture sets as above were infected for 2 days with csPc-preloaded (10 µM Pc 3.5) or control Leishmania, as indicated. Cells were then light-exposed and detached with trypsin-EDTA (Invitrogen) 1 day after light exposure. Cells were assessed by flow-cytometry for GFP fluorescence as a measure of infection. Note: The significant loss of GFP fluorescence due to Leishmania photolysis in the DCs of the experimental group, but not of the controls. (DOC) [file pone.0020786.s002.doc]

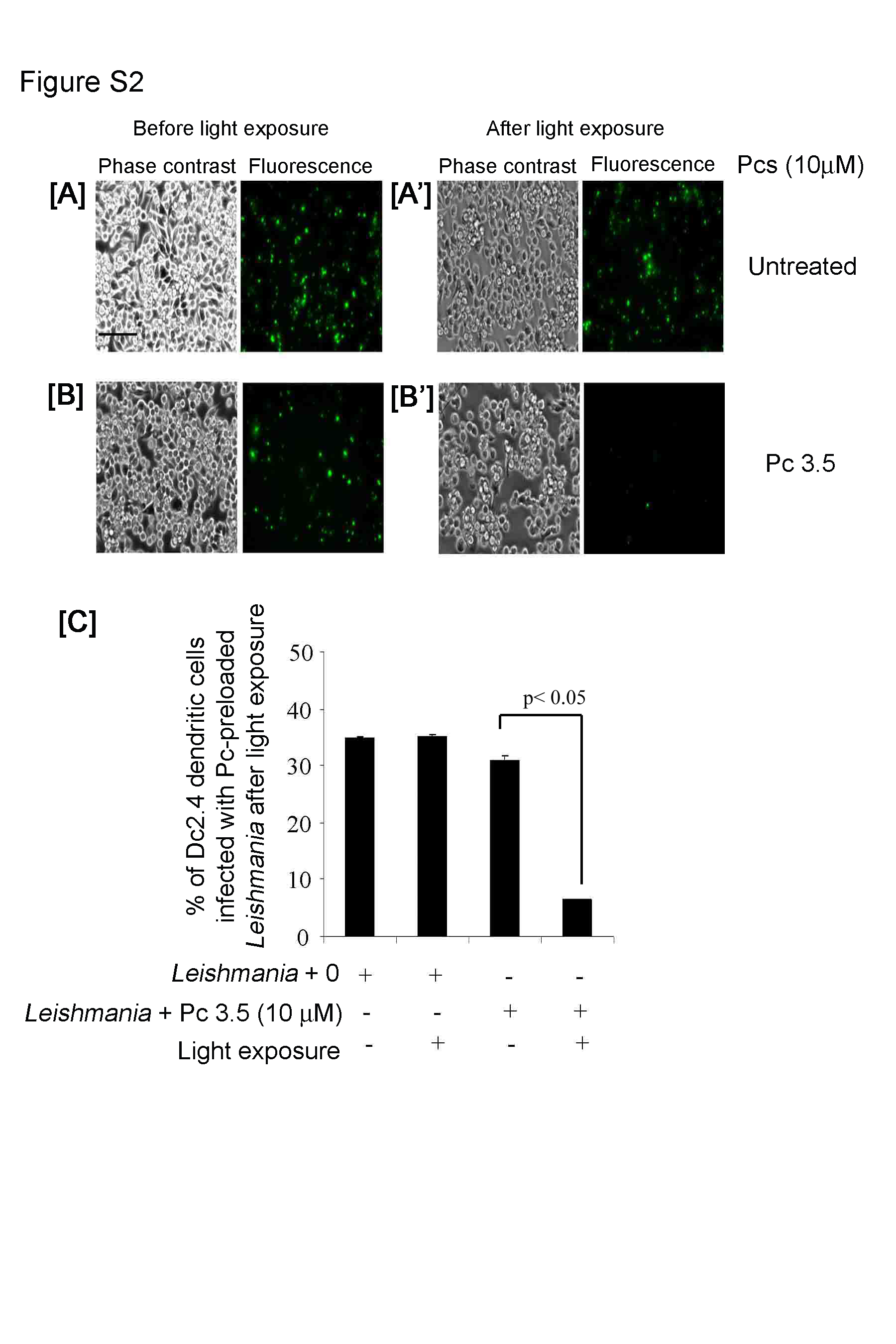


**Figure S2. Infection of DCs with csPc 3.5-loaded *Leishmania* and selective photolysis of the latter after illumination of infected cells**.

**[A-B, A’-B’] Phase contrast and fluorescence microscopic images of adherent DC 2.4 cells showing clearance of GFP-*Leishmania* infection**: GFP transfected *Leishmania* (see green fluorescence) were loaded overnight with or without 10 µM csPc 3.5 and used to infect DC 2.4 cells. Infected monolayers were washed to remove non-attached extracellular parasites and light-exposed. Cells were examined by phase contrast and GFP fluorescence microscopy immediately before [**A-B**] and 1 day after light exposure [**A’-B’**]. **Note:** The integrity of the DCs and the substantial clearance of *Leishmania* green fluorescence from all cultures, except the control infected with *Leishmania* without Pc pre-loading (**untreated**). Scale bar = 100 µm.

**[C]** **GFP flow cytometry of infected cells, showing substantial clearance of GFP *Leishmania* infection:** Similar culture sets as above were infected for 2 days with csPc-preloaded (10 µM Pc 3.5) or control *Leishmania*, as indicated. Cells were then light-exposed and detached with trypsin-EDTA (Invitrogen) 1 day after light exposure. Cells were assessed by flow-cytometry for GFP fluorescence as a measure of infection. **Note:** The significant loss of GFP fluorescence due to *Leishmania* photolysis in the DCs of the experimental group, but not of the controls.
